# Comparison of the Kelly's plication and TOT simultaneously with vaginal hysterectomy, on the incontinence, and sexual functions

**DOI:** 10.1590/S1677-5538.IBJU.2018.0019

**Published:** 2018

**Authors:** Neslihan Bayramoglu Tepe, Omer Bayrak, Huseyin Caglayan Ozcan, Mete Gurol Ugur, Ilker Seckiner

**Affiliations:** 1 Department of Obstetrics and Gynecology, University of Gaziantep, Gaziantep, Turkey; 2 Department of Urology, University of Gaziantep, Gaziantep, Turkey

**Keywords:** Pelvic Organ Prolapse, Urinary Incontinence, Quality of Life

## Abstract

**Purpose:**

To compare the effect of vaginal hysterectomy-anterior/posterior colporrhaphy with Kelly's plication(VH-KP), versus vaginal hysterectomy-anterior/posterior colporrhaphy-transobturator tape(VH-TOT) surgeries on incontinence, quality of life, and sexual functions in patients with pelvic organ prolapse(POP), and concurrent obvious stress urinary incontinence(SUI).

**Materials and Methods:**

Between 2013 and 2017, fifty patients treated with VH-KP(n = 25), and VH-TOT(n = 25) due to POP and SUI, were evaluated prospective consecutively. Age, parity, duration of urinary incontinence, and the daily pad use were recorded. Patients were filled “rinary Distress Inventory-6(UDI-6)”, “Incontinence Impact Questionnaire 7(IIQ-7)” and “Index of Female Sexual Function(IFSI)” questionnaire forms at preoperatively, and postoperative 6th month. No usage of pads was accepted as subjective cure rate. Intraoperative, and postoperative complications were noted.

**Results:**

There was no statistically significant difference between two groups, for the mean age of the patients, parity, duration of SUI, and the daily pad use, preoperatively (p > 0.05). Decreased UDI-6 scores, IIQ-7 scores and daily pad usage, and increased IFSF scores were found statistical significantly in each group, at the postoperative 6 th month (p < 0.05). However, VH-TOT group had higher improvement rates, on UDI-6 scores (69.5% vs 63.0%, p = 0.04). In addition, it was notable that the the rates of the patients had IFSF scores ≥ 25 was higher in VH-KP group (p = 0.05). Four (16%) patients had recurrent SUI in the VH-KP group (p = 0.039) and vaginal extrusion occurred in 2 (8%) patients in the VH-TOT group (p = 0.153), postoperatively.

**Conclusions:**

Although the effects of VH-TOT surgery are superior to conventional methods for incontinence and quality of life; negative effects on sexual functions are notable. In addition, although recurrence rates of TOT are low, complications such as vaginal extrusion are accompanied by drawbacks of mesh usage.

## INTRODUCTION

Pelvic organ prolapse (POP) is a common problem that occurs in half of multiparous women, and surgical treatment is required in one tenth of the patients (1–4). Accompanying urinary incontinence with symptomatic POP may affect sexual functions, may cause a decrease in self-confidence, and may lead to a poor relationship with the opposite gender (5) Some (11-45%) of the women with urinary incontinence may avoid sexual intercourse due to the fear of urinary incontinence (6–9).

Surgery of POP and stress urinary incontinence (SUI) enables the resolution of dysfunctions and helps to provide anatomic balance of organs. Although surgical success is aimed for anatomic recovery, subjective functional results, cosmetic appearance, quality of life, and sexual functions are required to be evaluated all together (6–9). SUI accompanying POP may be treated using conventional methods or other methods that require the use of mesh, such as the mid-urethral slings (MUS). Anterior / posterior colporrhaphy with Kelly's plication (CA-KP) is one of the conventional methods performed in the treatment of SUI. Although it is an old method and the success rate is lower in the long term, it has been a popular method in some clinics among gynecologists and urologists (10). However, after the description of transoburator tape (TOT) procedure in the treatment of SUI in 2001 by Delorme, TOT has been the gold standard in many centers as a minimally invasive method (11).

The number of studies comparing the different surgical techniques in POP accompanying SUI, and simultaneously investigating the effect of these surgical procedures on sexual functions are limited in the literature. We aimed to compare the effect of vaginal hysterectomy-anterior / posterior colporrhaphy with Kelly's plication (VHKP), versus vaginal hysterectomy-anterior / posterior colporrhaphy-transobturator tape (VH-TOT) operations on incontinence, quality of life, and sexual functions in patients with POP and concurrent obvious SUI.

## MATERIALS AND METHODS

### Study participants

After obtaining local ethics committee approval, 50 patients who were treated with VH-KP or VH-TOT due to POP and concurrent obvious SUI between 2013 and 2017, were evaluated prospectively and consecutively. In the scope of the study, VH-KP was performed in the first 25 patients, and VH-TOT (Unitape T^®^, Promedon, Cordoba, Argentina) was performed in the second 25 patients. Age, parity, duration of SUI, and the number of pads used daily were recorded. All patients were evaluated during the preoperative period using physical and gynecologic examinations, complete urine analysis, urine culture, stress and Q-type test, and urodynamics studies. POP was evaluated using the Baden-Walker Halfway System. All patients were selected from similar diagnostic group to create a more homogeneous group. Therefore, only patients with stage 4 POP (4: the maximum possible prolapse) were included in the study.

Patients with POP and concurrent obvious SUI, aged over 18 years with symptoms lasting more than 1 year, who were not willing pregnancy, who did not prefer organ protective methods, and in whom SUI was urodynamically demonstrated after prolapse reduction, were included in the study. Patients with neurogenic bladder, urinary system infection, and history of previous surgery due to POP and urinary incontinence, pelvic radiation history, whose residual urine after prolapse reduction was over 100 mL, and who had stage 1-3 prolapse according to the Baden-Walker Halfway System were excluded. In addition, patients who preferred pubovaginal sling surgery rather than mid-urethral sling surgery were not evaluated in the study.

### Efficacy evaluation

The efficacy of the surgical procedures was compared using the “Urinary Distress Inventory-6 (UDI-6)” and the “Incontinence Impact Questionnaire 7 (IIQ-7)”. Patients completed the UDI-6 and IIQ-7 forms preoperatively and at the postoperative 6^th^ month. Patient's daily pad usage was reexamined in the 6^th^ month, and improvement rates were calculated. Non usage of pads was accepted as subjective cure rate. In addition, sexually active patients in both groups completed “Index of Female Sexual Function (IFSF)” questionnaires. If the total IFSF score was lower than 25, it was accepted as sexual dysfunction (12–14).

## SURGICAL TECHNIQUES

Transobturator tape surgery was performed in accordance with the description of Delorme's, vaginal hysterectomies plus anterior / posterior colporrhaphy in accordance with the description of Heaney, and Kelly plication was performed using Howard Kelly's method. A vaginal pack was inserted in the vagina after surgery. Vaginal pack, and urethral catheter were removed on postoperative first day. Urethral catheters remained for several days in patients who had a post-voiding residual urine volume higher than 100 mL. Intraoperative and postoperative complications were recorded.

### Statistical analysis

The statistical package program Statistical Package for the Social Sciences (SPSS) 11 for Windows was used in statistical calculations. Data are described as arithmetic mean and standard deviation. The Chi-square distribution test was used for the calculation of categoric variables, and the Mann-Whitney U test was used for the comparison of means. “p values” lower than 0.05 were accepted as significant.

## RESULTS

Mean age in the VH-KP group was 62.20 ± 11.23 years, and 65.12 ± 11.18 years in the VH-TOT group (p = 0.460). There was no statistically significant differences between the two groups regarding parity, duration of SUI, and the number of daily pad use (p = 0.322, p = 0.585, p = 0.301, respectively) (Table-1).

**Table 1:** Demographic data of the patients and the operations.

	VH-KP	VH-TOT	p value
**Age (year)**	62.20 ± 11.23	65.12 ± 11.18	0.460
**parity (n)**	5.08 ± 2.21	5.76 ± 2.58	0.322
**Incontinence period (year)**	1.76 ± 1.42	2.12 ± 1.76	0.585
**pre-operative daily pad usage (n)**	1.92 ± 0.95	2.24 ± 1.2	0.310
**post-operative daily pad usage (n)**	0.4±0.64	0.36±0.48	0.935
**Sexual active patients (n, %)**	18 (72%)	18 (72%)	1

Decreased UDI-6 scores, IIQ-7 scores and daily pad usage, and increased IFSF scores were found statistical significantly in each group, at the postoperative 6^th^ month (Table-2). When we compared the groups for improvement rates, UDI-6 and IIQ-7 scores were observed higher in the VH-TOT group, but IIQ-7 scores were not calculated as statistical significantly (69.5% vs. 63.0%, p = 0.04, 67.3% vs. 57.8%, p = 0.182). Although subjective cure rates (68% vs. 64%, p = 0.765), and improvements on the daily pad usage (88.76% vs. 85.92%, p = 0.782) were higher in the VH-TOT group, there was no statistical difference. Eighteen (72%) patients in both groups were sexually active. It was notable that the rate of patients with IFSF scores ≥ 25 was higher in VH-KP group at the postoperative 6^th^ month (p = 0.05) (Table-3).

**Table 2:** Improvements on UDI-6, IIQ-7, IFSF scores, and daily pad use, at the postoperative 6^th^ month.

		Pre-operative	Post-operative	p value
**UDI-6 scores**				
	VH-KP	14.80 ± 2.5	5.92 ± 2.85	0.001*
	VH-TOT	10.60 ± 5.1	2.68 ± 1.65	0.001*
**IIQ-7 scores**				
	VH-KP	16.04 ± 5.01	6.96 ± 3.45	0.001*
	VH-TOT	12.84 ± 3.70	4.36 ± 0.99	0.001*
**Daily pad usage**				
	VH-KP	1.92 ± 0.95	0.4 ± 0.12	0.001*
	VH-TOT	2.24 ± 1.92	0.36 ± 0.09	0.001*
**IFSF scores**				
	VH-KP	13.27 ± 6.13	31.00 ± 5.69	0.001*
	VH-TOT	12.38 ± 7.63	27.33 ± 4.80	0.001*

**UDI-6** = Urinary Distress Inventory-6; **IIQ-7** = Incontinence Impact Questionnaire-7; **IFSI** = Index of Female Sexual Function; **VH-KP** = vaginal hysterectomy - anterior/ posterior colporrhaphy with Kelly's plication; **VH-TOT** = vaginal hysterectomy – anterior/posterior colporrhaphy - transobturator tape

* p values lower than 0.05 were accepted as significant.

**Table 3:** Improvements on UDI-6, IIQ-7, IFSF scores, and daily pad use, at the postoperative 6^th^ month.

	VH-KP	VH-TOT	p value
**UDI-6 scores (%)**	63.0 ± 14.22	69.52 ± 24.87	0.04*
**IIQ-7 scores (%)**	57.88 ± 23.32	67.32 ± 32.77	0.182
**daily pad usage (%)**	85.92 ± 21.42	88.76 ± 16.12	0.782
**subjective cure rates (%)**	64	68%	0.765
**IFSF scores (%)**	91.17 ± 17.97	78.72 ± 30.42	0.226
**IFSF scores ≥ 25, n (%)**	16 (88.8%)	11 (61.6%)	0.05

**UDI-6** = Urinary Distress Inventory-6; **IIQ-7** = Incontinence Impact Questionnaire-7; **IFSI** = Index of Female Sexual Function

* p values lower than 0.05 were accepted as significant.

No intraoperative complications were observed in either group. At the postoperative 6^th^ month, 4 (16%) patients had recurrent SUI in the VH-KP group (p = 0.039), and vaginal extrusion occurred in 2 (8%) patients in the VH-TOT group (p = 0.153).

## DISCUSSION

Sexual dysfunction rates increase in pelvic floor disorders such as POP and SUI, especially in older patients (15). Urinary incontinence is detected in 20-90% of women with POP, POP is found in 30-70% of women with UI, and sexual dysfunction is reported in 31-44% in women with POP and UI (16). Surgical intervention against POP and SUI provides higher rates of cure in incontinence compared with surgery for POP alone (17). In the present study, we performed VH for the treatment of POP, and KP or TOT procedures were additionally performed for SUI. Significant improvement in incontinence symptoms was observed at the postoperative 6^th^ month, on the UDI-6 scores, IIQ-7 scores, and daily pad usage in both groups (p = 0.001, p = 0.001, p = 0.001, respectively).

In a comprehensive meta-analysis that evaluated the effect of POP and UI surgeries on sexual functions, 21 studies were analyzed. Specific sexual symptoms were examined, or questionnaires were used in the evaluation of sexual functions in patients with UI. General sexual symptoms did not change after incontinence surgery in 18 studies (n = 1578 patients), in more than half (55.5%) of the patients. Sexual symptoms improved in 31.9% of the patients, and deterioration was reported in 13.1% of the patients. An analysis of the effects of mid-urethral sling surgeries [TOT, and tension-free vaginal tape (TVT)] on sexual functions in 16 studies comprising 1252 patients revealed that sexual functions did not change in 56.7%, improved in 33.9%, and worsened in 9.4%. The meta-analysis emphasized that improvement of sexual functions was 3-fold higher than the worsening of sexual functions with SUI surgery (18). However, in the present study, we observed that sexual function improved in both groups (p = 0.001, p = 0.001, respectively). Although it was not statistically significant, improvement in sexual function rates on IFSF questionnaires were found higher in the VH-KP group (91.1% vs. 78.7%, p = 0.226). In addition, the patients with IFSF scores ≥ 25 was higher in VH-KP group at the postoperative 6^th^ month (p = 0.05).

In a recent randomized study, Sohbeti S et al. randomized 60 patients into two groups (TOT vs. anterior colporrhaphy and Kelly's plication) who underwent SUI surgery. The cure rates in the first, 6^th^, and 12^th^ months in the TOT group were 86.7%, 80%, and 80%, respectively, and the cure rates in the anterior colporrhaphy with Kelly's plication group were 80%, 70%, and 66.7% (p = 0.68, p = 0.54, and p = 0.22, respectively). The authors reported that although there was no significant differences between the two surgical methods for the improvement of UI at the short-term follow-up, these outcomes might change in the long term (19). In the present study, decreased UDI-6 scores, IIQ-7 scores and daily pad usage, and increased IFSF scores were found statistical significantly in each group, at the postoperative 6^th^ month. But, VH-TOT group had higher improvement rates, on UDI-6 scores (69.5% vs. 63.0%, p = 0.04). According to current guidelines and recent studies, it is a reality that long-term recurrence of SUI will be lower in patients underwent TOT owing to the mesh usage (17).

However, improvements on sexual functions do not show concordance with improvements on SUI after surgical corrections of POP and / or SUI. It was remarkable to observe more recovery in UI symptoms in the VH-TOT group, and greater recovery in sexual functions in the VK-KP group. The finding of no change or deterioration in the sexual function could be due to changes in vaginal anatomy (mucosal damage, vaginal wall elevation, and narrowing), decreased sensation, libido loss, dyspareunia, anorgasmia, presence of high levels of residual urine, and de novo urgency after surgery (18, 20, 21). These results demonstrate the drawbacks of the mesh use. A recent meta-analysis by Jha et al. revealed that a significant increase in sexual function and decrease in dyspareunia was reported after POP surgery with conventional tissue repair (22). Mesh erosion / extrusions, which may be detected in 1% of patients after sling surgery, have a negative effect on sexual functions (18). In a study investigated the female sexual function after TOT, vaginal erosion was reported as 4.9%, de novo urgency as 4.9%, vesico-vaginal fistula as 1.2%, and urinary retention as 3.7% (23). In our study, vaginal extrusion was detected in 2 (8%) patients who underwent TOT, and mesh excision was performed. Although there was a significant improvement in sexual functions in the VH-KP group, recurrent SUI developed in 4 (16%) patients at the 6^th^ postoperative month.

There are limited studies comparing effects of surgeries on incontinence, quality of life, and sexual function in patients with POP, and concurrent obvious SUI. Therefore, we believe that the present study will contribute to the current literature. But, our study has certain limitations. The small sample size and short-term postoperative follow-up are the primary limitations. The current findings should be supported by prospective, randomized studies including wider patient series and long-term results.

## CONCLUSIONS

Although the improvement rates of VH-TOT surgeries are superior to conventional methods for SUI, and quality of life, the negative effects on sexual function are notable. Even though the SUI recurrence rates of TOT surgeries are lower, complications such as vaginal extrusion are known drawbacks associated with mesh usage. Therefore, in addition to treatment success, patients treated with mesh should be informed about its effects on sexual functions and potential complications. In addition, although it is an outdated method, conventional surgical methods such as Kelly's plication may be offered as an option for patients who request native tissue repair.
